# Dilatations des bronches chez les patients porteurs de bronchopneumopathie chronique obstructive au centre Tunisien: impact sur l’évolution et le pronostic

**DOI:** 10.11604/pamj.2020.37.200.24448

**Published:** 2020-10-29

**Authors:** Ahmed Ben Saad, Asma Migaou, Saousen Cheikh Mhamed, Nesrine Fahem, Naceur Rouatbi, Samah Joobeur

**Affiliations:** 1 Service de Pneumologie et d´Allergologie, Hôpital Universitaire Fattouma Bourguiba, Rue 1er juin, 5000 Monastir, Monastir, Tunisie

**Keywords:** Bronchopneumopathie chronique obstructive, BPCO, dilatation des bronches, exacerbation de maladie, hospitalisation, explorations fonctionnelles respiratoires, EFR, Chronic obstructive pulmonary, COPD, bronchial dilation, disease exacerbation, hospitalization, pulmonary function tests, PFT

## Abstract

**Introduction:**

les dilatations des bronches (DDB) semblent avoir un impact important sur l'histoire naturelle de la bronchopneumopathie chronique obstructive (BPCO). L'objectif de notre travail était d'étudier l'impact des DDB sur la sévérité, l'évolution et le pronostic des patients atteints de BPCO.

**Méthodes:**

c´est une étude rétrospective, monocentrique, analytique, s´étalant de 1995 à 2017, portant sur les dossiers de patients atteints de BPCO ayant eu un scanner thoracique durant la période du suivi. Nous avons comparé deux groupes (G) de patients: G1: BPCO avec DDB, G2: BPCO sans DDB.

**Résultats:**

notre étude a inclus 466 patients atteints de BPCO parmi eux 101 (21,6%) ayant des DDB associées à la BPCO. Les patients du G1 avaient un volume expiratoire maximum à la première seconde (VEMS) plus bas (G1: 1,21 L, G2: 1,37 L, p = 0,015), une capacité vitale forcée (CVF) plus basse (p=0,014), une PaO2 à l´état stable plus basse (p = 0,049), un nombre plus élevé des exacerbations aiguës (EA)/an (G1: 3,31, G2: 2,44, p = 0,001) et un nombre plus élevé d´hospitalisation en réanimation /an (p = 0,02). Lors des hospitalisations pour EA les patients du G1 étaient caractérisés par une PaO2 à l´admission plus basse (G1: 60 mmHg, G2: 63,7 mmHg, p = 0,023), une capnie plus élevée (p = 0,001), un recours plus fréquent à la ventilation non invasive (VNI) (p = 0,044) et à la ventilation mécanique invasive (p = 0,011). Les patients du G2 étaient caractérisées par une meilleure survie (p = 0,002).

**Conclusion:**

les DDB dans la BPCO sont un indicateur de mauvais pronostic, en particulier en termes de fréquence et de sévérité des exacerbations, d'obstruction sévère des voies respiratoires et de mortalité.

## Abstract (en)

**Introduction:**

bronchial dilations (BDs) seem to have a major role in the natural history of chronic obstructive pulmonary disease (COPD). The purpose of our study was to evaluate the impact of BDs on the severity and progression of COPD as well as on patients’ prognosis.

**Methods:**

we conducted a retrospective, single-center, analytical study over the period 1995- 2017. The study was based on data from the medical records of patients with COPD who had undergone chest CT scan during the follow-up period. We compared two groups (G) of patients: G1: COPD with BDs; G2: COPD without BDs.

**Results:**

our study included 466 patients with COPD. Among them 101 (21.6%) had BDs associated with COPD. G1 patients had lower maximum expiratory volume in the first second (FEV1) (G1: 1.21 L, G2: 1.37 L, p = 0.015), lower forced vital capacity (FVC) (p = 0.014), a lower PaO2 at steady state (p = 0.049), a higher rate of acute exacerbations (AE) per year (G1: 3.31, G2: 2.44, p = 0.001) and a higher rate of hospitalizations in the Intensive Care Unit per year (p = 0.02). G1 patients with AE receiving treatment in hospital had lower PaO2 3) on admission (G1: 60 mmHg, G2: 63.7 mmHg, p = 0.02 G2: 63.7 mmHg, p = 0.023), more elevated carbon dioxide (CO2) levels (p = 0.001) and were characterized by a higher use of non-invasive ventilation (NIV) (p = 0.044) and invasive mechanical ventilation (p = 0.011). G2 patients had better overall survival (p = 0.002).

**Conclusion:**

bronchial dilatations are an indicator of poor prognosis in patients with chronic obstructive pulmonary disease, expecially because of the higher rate and severity of exacerbations, airway obstructions and mortality.

## Introduction

La bronchopneumopathie chronique obstructive (BPCO) est définie comme une maladie commune qu´on peut prévenir et traiter, caractérisée par des symptômes respiratoires persistants et une limitation persistante des débits aériens dues à des anomalies des voies aériennes et/ou alvéolaires souvent causées par une exposition significative à des particules nocifs ou gaz [1]. Le diagnostic repose sur l´existence d´un trouble ventilatoire obstructif qui est défini par un rapport volume expiratoire maximum seconde (VEMS) / capacité vitale forcée (CVF) post bronchodilatation < 70% [1]. Le tabagisme est le principal facteur de risque de BPCO [1, 2]. En effet, environ 15 à 50% des fumeurs chroniques développent la BPCO [3, 4]. La BPCO est l´un des principaux problèmes de santé publique, non seulement en raison de sa prévalence élevée et les coûts de santé associés élevés, mais aussi en raison de la morbidité et la mortalité résultantes élevées et la diminution de la qualité de vie liée à la santé [5].

Actuellement, la BPCO est considérée comme la troisième cause de mortalité mondiale [6]. Les comorbidités sont courantes au cours de la BPCO et se sont révélées être associées à la mortalité, à une mauvaise qualité de vie et au mauvais état de santé [7]. Les dilatations des bronches (DDB) ou bronchectasies font partie des comorbidités associées à la BPCO. En effet, les DDB ont été considérées pour la première fois comme une des comorbidités de la BPCO dans The Global Initiative for Chronic Obstructive Lung Disease (GOLD) 2014 Guidelines [8]. Elles sont définies par une augmentation permanente et irréversible du calibre des bronches. Le diagnostic de DDB se fait par le scanner thoracique. La prévalence des DDB chez les patients atteints de BPCO a été analysée dans plusieurs études, avec des résultats différents allant de 4 à 72% [9, 10].

L'identification des DDB chez les patients BPCO a permis de définir un phénotype clinique particulier caractérisé par des signes cliniques plus sévères, des infections bronchiques chroniques, des exacerbations plus fréquentes et un mauvais pronostic. Une association causale n'a pas encore été prouvée, mais il est plausible que la BPCO, et en particulier le phénotype fréquent exacerbateur de la BPCO, puisse être la cause des DDB sans autre étiologie connue, au-delà d'une simple association ou comorbidité. La présence d´un phénotype de bronchite chronique peut également accroître le risque d´infection bronchique chronique et d´exacerbations infectieuses récurrentes, qui perpétuent le cercle vicieux de l´infection, de l´inflammation et de la destruction tissulaire. L'étude de la relation entre la BPCO et les DDB pourrait avoir des implications cliniques importantes, car les deux maladies ont des approches thérapeutiques différentes et complémentaires. Il existe peu de données africaines relatives à la relation entre les DDB et la BPCO. L´objectif de notre étude était d´évaluer l´impact des DDB sur la sévérité, l'évolution et le pronostic des patients atteints de BPCO.

## Méthodes

### Type d´étude

Il s´agit d´une étude rétrospective, monocentrique, analytique portant sur les dossiers des patients suivis pour BPCO au service de Pneumologie et d´Allergologie à l´Hôpital Universitaire Fattouma Bourguiba de Monastir entre Janvier 1995 et Décembre 2017.

### Critères de sélection des dossiers

Les patients inclus dans cette étude étaient les malades atteints de BPCO selon la définition du GOLD [1]: la présence d´une symptomatologie respiratoire chronique (toux, expectorations, dyspnée d´effort), et/ou d´une histoire d´exposition aux facteurs de risque de BPCO; la présence d´un trouble ventilatoire obstructif à la spirométrie définie par un rapport VEMS/CVF < 70% post-bronchodilatation.

**Figure 1:**
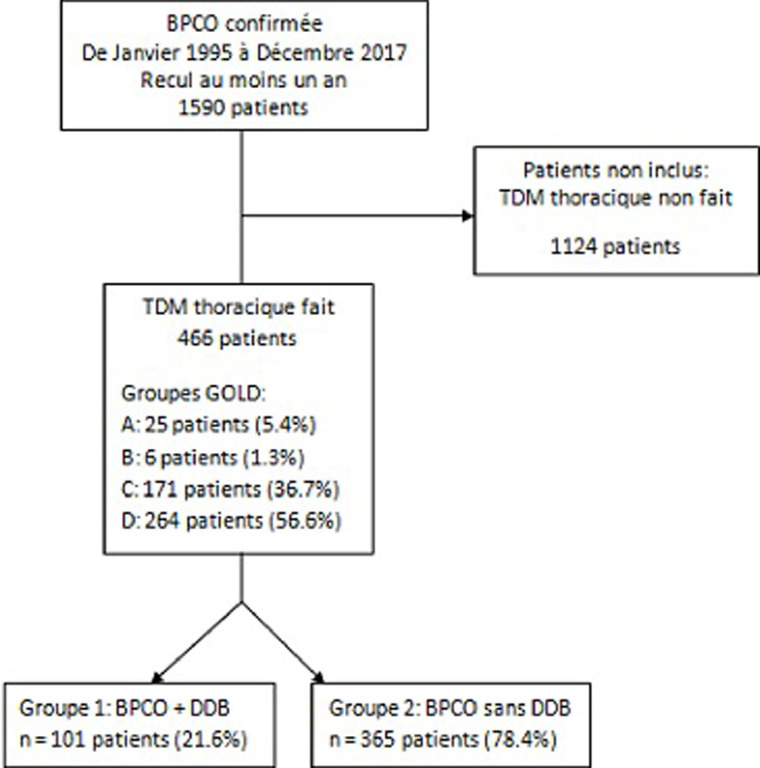
diagramme de flux des patients de l'étude

Les sujets inclus dans notre étude étaient des patients atteints de BPCO diagnostiquée et suivie depuis au moins un an et ayant eu un scanner thoracique durant la période du suivi. Nous avons vérifié la présence ou non des DDB associées à la BPCO (Figure 1). Nous n´avons pas inclus dans cette étude les patients atteints d´affections respiratoires chroniques comportant un trouble ventilatoire obstructif permanant et ne rentrant pas dans le cadre de la BPCO: l´asthme bronchique dans sa forme chronique avec dyspnée continue; le syndrome de chevauchement asthme-BPCO (Asthma-COPD Overlap Syndrome) selon les recommandations de GINA 2019 [11]; les bronchiolites chroniques de l´adulte; Certaines formes de dilatations des bronches ayant évoluées vers un trouble ventilatoire obstructif. Les patients porteurs de cancer bronchique étaient exclus de l´étude de la survie du fait que la survie de ces patients est plutôt liée à l´évolution de la pathologie néoplasique.

### Evaluation des paramètres cliniques, fonctionnels respiratoires, évolutifs et pronostiques de la BPCO

**Evaluation des paramètres cliniques et fonctionnels respiratoires:** différents paramètres de la BPCO ont été évalués, comportant des paramètres cliniques (les comorbidités, la dyspnée) et des paramètres fonctionnels respiratoires à l'état stable (exploration ventilatoire, gazométrie sanguine, classification de BPCO selon GOLD).

**Classification de la BPCO:** nous avons considéré la dernière classification du GOLD 2020 pour classer nos patients en groupes [1]. En effet cette classification prend en considération la symptomatologie selon le niveau de dyspnée déterminé par l´échelle mMRC et le nombre d´exacerbations aiguës (EA). D´autre part on a classé nos patients BPCO selon la sévérité de l´obstruction bronchique de GOLD 1 à 4 [1].

**Evaluation de paramètres évolutifs:** différents paramètres évolutifs ont été évalués comportant le nombre d'exacerbations aiguës par an, le nombre d'hospitalisations en pneumologie et en réanimation par an, l'évolution vers l'insuffisance respiratoire chronique (IRC) et le recours à l'oxygénothérapie de longue durée (OLD). Pour les EA, différents paramètres de sévérité ont été évalué comportant la gazométrie sanguine à l´admission, le syndrome inflammatoire biologique à l´admission (nombre de globules blancs, C-réactive protéine (CRP)), la durée d´hospitalisation et l´évolution de l´exacerbation sous traitement (recours à la ventilation non invasive (VNI), recours à la ventilation mécanique invasive (VMI), hospitalisation en réanimation, délai de la prochaine EA sévère). Nous avons déterminé la moyenne des différentes variables quantitatives.

**Pronostic de la BPCO:** les données concernant la survie étaient recueillies à partir des dossiers pour les patients décédés à l´hôpital. Pour les autres patients, les informations sur la survie étaient recueillies par contact téléphonique.

### Impact des DDB sur la sévérité, l´évolution et le pronostic de la BPCO

Afin d´évaluer l'impact des DDB sur la sévérité, l´évolution et le pronostic de la BPCO, nous avons défini 2 groupes de patients selon la présence ou l´absence de DDB: Groupe 1 (G1): BPCO avec DDB; Groupe 2 (G2): BPCO sans DDB. Nous avons comparé les différents paramètres de sévérité de BPCO, l´évolution et le pronostic entre les deux groupes.

### Analyse statistique

Les données ont été saisies et analysées grâce au logiciel SPSS (Version 20). Les variables quantitatives sont exprimées en moyennes ± déviations standards (DS). Les variables qualitatives sont exprimées en taux. La comparaison des variables qualitatives a été faite au moyen du test de Chi2. La comparaison des variables quantitatives a été faite au moyen du test de Student. Une valeur (p) inférieure à 0,05 était considérée comme statistiquement significative. Les facteurs indépendants caractérisant l´association BPCO-DDB ont été déterminés par analyse multivariée. Les facteurs retenus pour l´analyse multivariée étaient tous ceux ayant une valeur (p) inférieure à 0,2 lors des analyses univariées. Dans l´étude de l´influence de l´association des DDB à la BPCO sur la survie, on s´est basé sur l´analyse par la méthode de Kaplan Meier avec les tests statistiques de Log-rank et de Breslow.

## Résultats

### Caractéristiques de la population étudiée

Notre étude a inclus 466 patients atteints de BPCO parmi eux 101 (21,6 %) ayant des DDB associées à la BPCO. L'âge moyen était de 64,9 ans avec une nette prédominance masculine (97,2%). Le nombre moyen d'EA/an était de 2,63. Le délai moyen de suivi de nos patients était de 4,2 ans (Tableau 1).

**Tableau 1:** caractéristiques démographiques et cliniques des patients BPCO avec et sans DDB à l'état stable

Characteristics	BPCO	BPCO avec DDB	BPCO sans DDB	p
Patients, n (%)	466	101 (21.7)	365 (78.3)	-
Genre masculin, n (%)	453 (97.2)	98 (97)	355 (97.3)	**0.90**
Age (années) ±DS	64.9± 11.6	63.9±11.8	65.2±11.6	0.29
Tabagisme: actif et passif, n (%)	452 (97)	96 (95)	356 (97.5)	0.19
Paquets-année ±DS	58±29	54±32	60±28	0.11
IMC (kg/m2) ±DS	23.1±5.3	23.6±4.9	22.9±5.4	0.27
Comorbiditiés ≥1	407 (87.5)	94 (93.1)	313 (86)	0.06
mMRC≥2, n (%)	238 (51.1)	51 (50.5)	187 (51.2)	0.89
CVF (L)	2.1	1.94	2.16	**0.01**
VEMS post bronchodilation (L)	1.34	1.21	1.37	**0.01**
VEMS/CVF post bronchodilation (%)	57.9±10	57±10	58.1±10.1	0.34
GOLD III et IV, n (%)	287 (61)	70 (69.3)	217 (59.5)	**0.04**
PaO2 (mmHg)	71.7	69.4	72.3	**0.04**
PaCO2 (mmHg)	39.1	41.5	38.5	**<10-3**
**corticostéroïde inhalé, n (%)**	310 (66.5)	69 (68.3)	241 (66)	0.66
n EABPCO/an	2.63	3.31	2.44	**10-3**
n EA sévère de BPCO/an	1.06	1.23	1.02	0.16
**Hospitalisation en réanimation** (n/patient/an)	0.13	0.25	0.1	**0.02**
Insuffisance respiratoire chronique, n (%)	183 (39.3)	47 (46.5)	136 (37.3)	0.09
Oxygène à domicile, n (%)	64 (13.7)	21 (20.8)	43 (11.8)	**0.02**

BPCO: broncho-pneumopathie chronique obstructive, DDB: dilatations des bronches, n: nombre, DS: déviation standard, IMC: indice de masse corporelle, mMRC: modified Medical Research Council dyspnea scale, CVF: capacité vitale forcée, VEMS: volume expiratoire maximum seconde, EA: exacerbation aiguë.

### Impact des DDB sur la sévérité, l´évolution et le pronostic de la BPCO

Afin d´évaluer l'impact des DDB sur la sévérité, l´évolution et le pronostic de la BPCO, nous avons défini 2 groupes de patients selon la présence ou l´absence de DDB: Groupe 1 (G1): BPCO avec DDB (101 patients, 21,6%). Groupe 2 (G2): BPCO sans DDB (365 patients, 78,4%).

### Impact des DDB sur la BPCO à l'état stable

Le G1 était caractérisé par une CVF significativement plus basse par rapport au G2, avec un VEMS plus bas, une PaO2 plus basse, une capnie plus élevée, un nombre d'EABPCO/an plus élevé, avec plus d'hospitalisation en unité de soins intensifs et un recours plus fréquent à l'oxygène longue durée (Tableau 1).

### Impact sur les paramètres de sévérité des EA sévères de BPCO

Les EA sévères des patients du G1 étaient caractérisés par une hypoxie plus importante, une capnie plus élevée, avec plus de recours à la VNI et à la VMI (Tableau 2).

**Tableau 2:** caractéristiques des exacerbations aiguës sévères chez les patients BPCO avec et sans DDB

	BPCO avec DDB	BPCO sans DDB	p
CRP (mg/L)	89	81	0.51
PaO2 (mmHg)	60	63.7	**0.02**
PaCO2 (mmHg)	43.4	39.9	**10-3**
VNI (%)	17.8	10.4	**0.04**
VMI (%)	14.9	6.9	**0.01**
Durée moyenne d´hospitalisation (jours)	10.4	11.6	0.14

BPCO: broncho-pneumopathie chronique obstructive, DDB: dilatations des bronches, CRP: C-reactive protein, VNI: ventilation non invasive, VMI: ventilation mécanique invasive.

### Impact sur la survie

**Figure 2:**
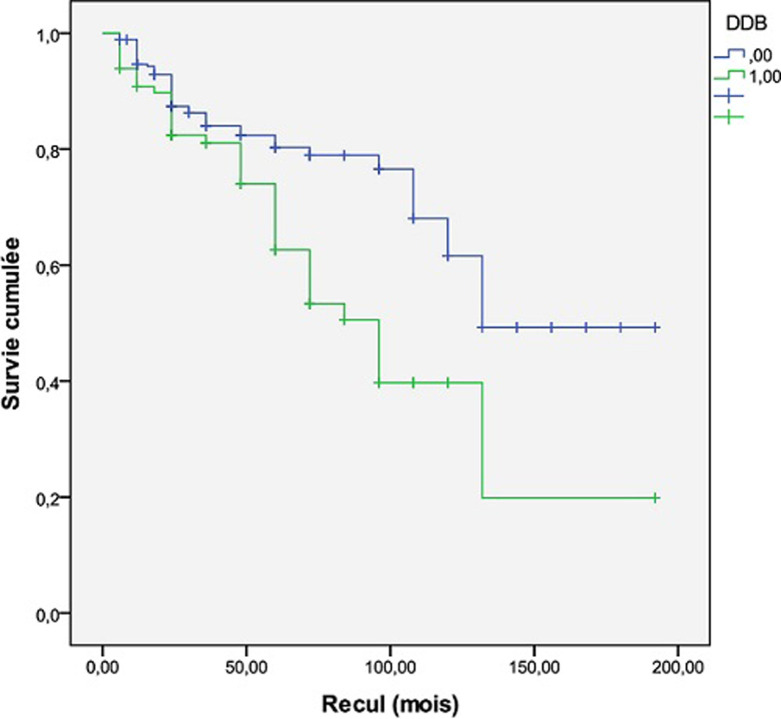
impact des DDB sur la survie des patients porteurs de BPCO

L´étude de la survie par la méthode de Kaplan Meier avec le test de Log-rank et le test de Breslow a mis en évidence une meilleure survie chez les patients BPCO sans DDB. En effet la médiane de survie était de 132 mois chez les patients BPCO sans DDB et de 96 mois chez les patients ayant une DDB associée à la BPCO avec une différence statistiquement significative (Log-rank: p = 0,002, Breslow: p = 0,036) (Figure 2).

### Facteurs indépendants caractérisant l´association BPCO-DDB

Les facteurs indépendants caractérisant l´association BPCO-DDB étaient la capnie à l´état stable (OR 1.057, 95% IC 1.010-1.105, p = 0,01) et le nombre d´EA de BPCO par an (OR 1.227, 95% IC 1.087-1.384, p = 0,01) (Tableau 3).

**Tableau 3:** facteurs indépendants caractérisant l'association BPCO-DDB

	p	OR	IC (95%)
PaCO2	**0.01**	1.057	1.010-1.105
**Nombre** EA / an	0.01	1.227	1.087-1.384

BPCO: broncho-pneumopathie chronique obstructive, DDB: dilatations des bronches, OR: Odds Ratio, IC: Intervalle de Confiance, EA: exacerbation **aiguë**

## Discussion

A travers cette étude rétrospective portant sur les dossiers de 466 patients atteints d´une BPCO ayant eu un scanner thoracique durant la période du suivi, nous avons étudié l´influence des DDB sur la sévérité, l'évolution et le pronostic de la BPCO chez ces patients. Nous avons montré que les DDB ont un impact péjoratif sur la sévérité de la BPCO à l´état stable (paramètres fonctionnels respiratoires, classification de la BPCO) ainsi que sur son évolution (IRC, OLD, EA, hospitalisation) et son pronostic (survie). Il existe peu de données africaines relatives à la relation entre la BPCO et les DDB.

La BPCO est caractérisée par la survenue d´exacerbations aiguës qui ont un important impact sur le pronostic de la maladie [12-14]. D'autre part, plusieurs études montrent que les DDB coexistent avec la BPCO dans 4 à 72% des cas [9, 10, 15]. Cela pourrait aggraver le cercle vicieux infectieux caractérisant les deux pathologies. Bien que l'association BPCO-DDB a été reconnue, il n'est pas clair s´il y a un lien de causalité entre avoir une BPCO et le développement des DDB.

### Impact sur la BPCO à l´état stable

**Impact sur les paramètres cliniques:** nous n'avons pas retrouvé un impact significatif des DDB sur la sévérité de la dyspnée à l´état stable. Dans la littérature l´impact des DDB sur la sévérité de la dyspnée est variable selon les études (17-20). Les données de l´étude de Zhang *et al*. [16] et celle de Kawamatawong *et al*. [17] sont en concordance avec notre résultat concernant la dyspnée de base. Alors que, le travail de Jin *et al*. [18] et celui de Martínez *et al*. [19] ont montré un impact péjoratif significatif des DDB sur la sévérité de la dyspnée à l´état stable chez les patients atteints de BPCO.

**Impact sur les paramètres fonctionnels respiratoires:** à travers notre étude, nous avons montré que les DDB ont un impact péjoratif sur différents paramètres fonctionnels respiratoires à l´état stable. Les données de la littérature relatives à l´impact des DDB sur les paramètres fonctionnels respiratoires de BPCO à l´état stable sont variables selon les études [20-23]. L´étude de Dou *et al*. [21] a montré un impact péjoratif significatif des DDB sur les paramètres ventilatoires (VEMS, CVF) à l´état stable. L´étude de Jin *et al*. [18] a objectivé un impact péjoratif significatif des DDB sur le VEMS et sur la sévérité du trouble ventilatoire obstructif (classification GOLD). De même, l´étude de Zhang [16] a montré cet impact sur le VEMS tandis que l´impact sur la sévérité du trouble ventilatoire obstructif n´était pas significatif. Dans l´étude de Dou *et al*. [22], la PaCO2 était significativement plus élevée dans le groupe BPCO avec DDB. Alors que l´étude de Mao *et al*. [23] n´a pas objectivé cet impact péjoratif sur la gazométrie sanguine. Nous avons retrouvé que la PaCO2 est un facteur indépendant caractérisant l´association BPCO-DDB. En effet, l'aggravation de la PaCO2 se voit souvent dans les formes avancées de la BPCO avec une fonction respiratoire altérée.

### Impact sur les paramètres évolutifs

**Insuffisance respiratoire chronique:** environ 2/5 (39,3%) de nos patients étaient au stade d´IRC avec 13,7% des cas sous OLD. Le pourcentage des patients nécessitant l´OLD était significativement plus élevé dans le groupe BPCO avec DDB par rapport au groupe BPCO sans DDB (G1: 20,8%, G2: 11,8; p = 0,02). Ce résultat est comparable avec celui de l´étude de Crisafulli *et al*. [24] où le recours à l´OLD était plus fréquent dans le groupe BPCO avec DDB (36% versus 23%; p = 0,01) et avec celui de Martínez [19] (34,8% versus 11,6%; p = 10^-3^).

**Exacerbations aiguës:** l'histoire naturelle de la BPCO est ponctuée par des exacerbations qui sont responsables d'une grande proportion des coûts de soins de santé [25, 26]. A travers notre travail, nous avons montré que le nombre des EA / an était significativement plus élevé dans le groupe BPCO avec DDB. Dans la littérature, plusieurs études montrent que les DDB augmentent le risque et le nombre des EA chez les patients atteints de BPCO [9, 20, 27]. L´étude de Martínez [19] est un travail prospectif fait entre 2004 et 2007 en Espagne. Il avait pour objectif d´évaluer la valeur pronostique des DDB chez les patients avec BPCO modérée à sévère. Cette étude a inclus 201 patients, parmi eux 115 patients avaient des DDB associées. Elle a montré que le nombre total des consultations aux urgences était significativement plus élevé dans le groupe BPCO avec DDB (1,88 versus 1; p = 0,002) ainsi que le nombre des EA sévères (1,12 versus 0,51; p = 0,002). L´étude de Kawamatawong [17] a inclus 72 patients atteints de BPCO, parmi eux 34 ayant des DDB associées. L'étude avait pour objectif d´évaluer l´impact des DDB sur la fréquence et la sévérité des EA de BPCO. Elle a montré que 62,07% des patients parmi le groupe BPCO avec DDB avaient au moins 2 EA ou une hospitalisation par an versus 37,93 % pour groupe BPCO sans DDB (p = 0,03). En effet, les DDB augmentent le risque des infections pulmonaires et de colonisation bactérienne en particulier par *Haemophilus influenzae* et *Pseudomonas Aeruginosa* [9, 20, 27-30].

Ainsi, les DDB favorisent les infections respiratoires qui sont les principales causes des EA et sachant que le meilleur prédicteur des exacerbations est une histoire d'exacerbations [31], les DDB engendrent ainsi une perpétuation des EA chez les patients atteints de BPCO. Notre étude a montré que le nombre d´EA / an est un facteur indépendant caractérisant l´association BPCO-DDB.

### Hospitalisations

Nous avons retrouvé que le nombre d´hospitalisation en pneumologie et en réanimation était plus élevé dans le groupe BPCO avec DDB avec une différence significative pour le nombre d´hospitalisation en réanimation (G1: 0,25/patient/an, G2: 0,1/patient/an; p = 0,02). L´étude de Su *et al*. [32] qui a inclus 4152 patients atteints de BPCO parmi eux 831 ayant des DDB associées, a montré que comparé au groupe de BPCO sans DDB, le groupe BPCO avec DDB avait significativement plus des EA (p < 10^-3^), des visites aux services des urgence (p < 10^-3^), des admissions à l'hôpital (p < 10^-3^), des admissions en réanimation (p < 10^-3^) et des jours d´hospitalisation (p < 10^-3^).

### Impact sur la sévérité des EA

Nous avons montré que les DDB ont un impact péjoratif significatif sur certains paramètres de sévérité des EA tel que les données de la gazométrie sanguine à l´admission (PaO2: p = 0,023, PaCO2: P = 10-3), le recours à la VNI (p = 0,04) et à la VM (p = 0,01). L´étude de Minov [33] qui a inclus 54 patients BPCO dont la moitié d´eux avait des DDB associées et qui avait pour objectif l´évaluation de la fréquence et la gravité des EA bactériennes chez les patients atteints de BPCO avec DDB, a montré que les DDB ont une influence négative sur la fréquence des EA, la durée des EA et le délai de la prochaine EA. En effet, le nombre moyen des EA était significativement plus élevé dans le groupe BPCO avec DDB (2.9 ± 0,5 versus 2,5 ± 0,3; p < 10^-3^) ainsi que la durée moyenne des EA exprimée en jours nécessaires à la guérison ou à l'amélioration clinique, c’est-à-dire résolution complète des symptômes ou retour des symptômes à leur état de base (6,9 ± 1,8 versus 5,7 ± 1,4; p = 0,01). Le délai de la prochaine EA était plus court dans le même groupe (56,4 ± 17,1 jours versus 67,2 ± 14,3 jours; p = 0,01). L´étude de Crisafulli *et al*. [24] qui a inclus 449 patients atteints de BPCO parmi eux 160 ayant des DDB associées et dont l´objectif était d´étudier l´impact des DDB sur les patients hospitalisés pour EA de BPCO, a montré que la PaCO2 à l´admission était significativement plus élevée dans le groupe BPCO sans DDB (48,8 versus 45,9; p = 0,003). Le groupe BPCO avec DDB avait moins d´hospitalisation en réanimation (7% versus 15%; p = 0,01) et un moindre recours à la VNI (15% versus 25%; p = 0,01). Il n´y avait pas une différence significative concernant le syndrome inflammatoire biologique à l´admission (globules blancs: p = 0,58, CRP: p = 0,31), le recours à la VM (p = 0,83) et la durée d´hospitalisation (p = 0,86) dans cette étude.

### Impact sur la survie

Notre travail a montré que les DDB ont un impact péjoratif sur le pronostic de patients atteins de BPCO. En effet, nous avons mis en évidence une meilleure survie chez les patients BPCO sans DDB comparativement à ceux ayant des DDB associées à la BPCO (Log-rank: p = 0,002, Breslow: p = 0,036). Notre résultat est en concordance avec les données de nombreuses études de la littérature [19, 23, 29]. L´étude de Mao *et al*. [23] qui a inclus 896 patients atteints de BPCO parmi eux 311 avaient des DDB associées et qui avait pour objectif l´évaluation des caractéristiques cliniques et la valeur pronostique des DDB chez les patients BPCO a montré que le taux de survie était moindre dans le groupe BPCO avec DDB (p = 0,02). L´étude de Du *et al*. [29], une métanalyse qui a inclus 14 études comportant en totalité 5329 patients atteints de BPCO parmi eux 1572 (29,5%) ayant des DDB associées a conclu que la présence de DDB chez les patients BPCO augmente le risque de mortalité (OR 1.96, 95% CI, 1.04-3.70). L´impact péjoratif des DDB sur la survie des patients BPCO pourrait être expliqué par différents facteurs: les EA, le déclin accéléré de la fonction respiratoire, et l'inflammation systémique et pulmonaire.

Parmi les points forts de notre étude nous citons l'absence d´études similaires dans la littérature africaine et le nombre de cas inclus dans cette étude (N = 466). Cependant, elle n´est pas sans limites. En effet, notre travail est rétrospectif, donc on ne peut pas maitriser toutes les données cliniques et paracliniques en particulier les données bactériologiques. Nous citons aussi la période prolongée de l'étude et la différence de taille des deux groupes. D´autre part, le scanner thoracique n´a pas été fait à la recherche des DDB dans la majorité des cas mais il a été demandé pour d´autres indications et certainement, il y´avait une différence de résolution scanographique vue le développement technologique entre 1995 et 2017 ce qui pourrait sous diagnostiquer les DDB chez les patients BPCO.

## Conclusion

A travers ce travail, les DDB semblent avoir un impact péjoratif sur la sévérité (fonction respiratoire), l´évolution (exacerbations, hospitalisations, et insuffisance respiratoire chronique...) et le pronostic de la BPCO. Cela a des implications importantes pour notre pratique et notre approche actuelles à l'évaluation et à la gestion de la BPCO. La coexistence des DDB doit être considérée comme un phénotype pathologique particulier de la BPCO, qui est un indicateur de mauvais pronostique. D´autres études sont nécessaires pour une meilleure compréhension des mécanismes physiopathologiques régissant cette association, des particularités de ce phénotype et des implications thérapeutiques.

### Etat des connaissances sur le sujet

- Vu la large utilisation du scanner thoracique actuellement, la découverte des DDB chez les patients BPCO est de plus en plus fréquente avec une relation parfois conflictuelle de ces deux entités dans la littérature;
- L'identification des DDB chez les patients BPCO a permis de définir un phénotype clinique particulier caractérisé par des signes cliniques plus sévères, des infections bronchiques chroniques, des exacerbations plus fréquentes et un mauvais pronostic.

### Contribution de notre étude à la connaissance

- A notre connaissance, il s'agit du premier travail étudiant cette association dans une population africaine avec ses particularités relatives aux BPCO et aux DDB ;
- Nous avons montré que les DDB ont un impact péjoratif sur la sévérité de la BPCO à l´état stable (paramètres fonctionnels respiratoires, classification de la BPCO) ainsi que sur son évolution (IRC, OLD, EA, hospitalisation) et son pronostic (survie);
- Perspective: une prise en charge globale du patient BPCO tenant en considération la présence des DDB et rentrant dans le cadre d'un plan d'action bien défini s'avère nécessaire.

## Remerciements

Nous tenons à remercier tout le personnel hospitalier qui a bien renseigné et a archivé les dossiers que nous avons utilisé pour cette étude.
